# The Risk of Injury in Wrist Arthroscopy Portals: A Cadaveric Study

**DOI:** 10.7759/cureus.49702

**Published:** 2023-11-30

**Authors:** Georgios Antonoglou, Aristeidis Vrettakos, Dimitrios Varvarousis, Panagiotis Kanavaros, Theodore Troupis, George K Paraskevas, Chrysanthos Chrysanthou, Elpida Apostolidi, Alexandros Poutoglidis

**Affiliations:** 1 Anatomy, University of Ioannina, Ioannina, GRC; 2 Orthopaedics, Agios Pavlos General Hospital of Thessaloniki, Thessaloniki, GRC; 3 Orthopaedics, University General Hospital of Ioannina, Ioannina, GRC; 4 Anatomy, National and Kapodistrian University of Athens, Athens, GRC; 5 Orthopaedics, Aristotle University of Thessaloniki, Thessaloniki, GRC; 6 Anatomy and Surgical Anatomy, Aristotle University of Thessaloniki, Thessaloniki, GRC

**Keywords:** volar wrist, dorsal wrist, cadaveric study, wrist arthroscopy, wrist arthroscopy portals

## Abstract

During wrist arthroscopy, the wrist joint can be visualized from almost every perspective through a combination of standard dorsal and volar arthroscopic portals. This cadaveric study aims to compare all wrist portals described in terms of their safety in order to rank them according to the distance from the nearest structure at risk for arthroscopic wrist procedures. Twenty-nine cadaveric formalin-embellished upper limbs were examined. Needles were inserted at dorsal and volar portal sites to perform the measurements. During the subsequent dissection, distances were measured as the shortest possible distance from the nearest structure at risk for each portal. Safe zones were determined for all portals, and the safety classification of arthroscopic wrist portals was proposed, ranking them from the safest to the most perilous. Applying the proposed safety classification to arthroscopic practice, wrist arthroscopy can be performed with a lower risk of iatrogenic complications arising from the implementation of the wrist portals.

## Introduction

The constantly evolving wrist arthroscopy is applied to diagnose and treat almost all the spectrum of wrist pathologies [1-2]. The development of smaller and more efficient equipment made wrist arthroscopy a valuable tool for repairing ligamentous injuries and triangular fibrocartilage complex lesions [3]. Other applications of wrist arthroscopy include carpal fusion, carpal coalition, accessory bones in this region, the treatment of carpal fractures, the removal of loose bodies, the debridement of chondral lesions, the irrigation of the wrist in cases of sepsis, and the evaluation of unexplained carpal wrist pain or trauma. Surgical treatment of fusion has revealed pain relief, restored mobility, and acceptable postoperative wrist motion effects [4-6].

The wrist can be thought of as a box and may be visualized from almost every perspective, having in mind the "box concept" [1]. Particularly, through a combination of arthroscopic dorsal and volar portals, it is possible to encircle the wrist using viewing and working portals from every direction [7]. The standard dorsal portals include radiocarpal 1-2, 3-4, 4-5, 6-radial (6-R), 6-ulnar (6-U), midcarpal radial (MC-R), and midcarpal ulnar (MC-U) portals [8]. The volar approach utilizes volar radial (VR) and volar ulnar (VU) portals previously described by Slutsky, alongside volar central radiocarpal (VC-RC) and volar central midcarpal (VC-MC) portals described by Corella et al. [9-12].

The purpose of this cadaveric study is to compare all the dorsal and volar wrist arthroscopy portals regarding their safety. Eventually, an attempt was made to propose a new classification based on the risk of every portal.

## Materials and methods

A systematic prospective study was conducted on consecutive fresh frozen cadaveric upper limbs in the laboratory of anatomy, histology, and embryology at the University of Ioannina, Ioannina, Greece, from 2019 to 2022. The dorsal and volar wrist arthroscopy portals were examined to evaluate their safety. The limbs were severed proximally to the humerus. Our study included 29 upper limbs. The study was approved by the institutional review board (IRB) of our department (number: 3372/15-07-2014).

Limbs were positioned in the prone and supine positions to evaluate the dorsal and volar portals, respectively. The elbow was fixed to a 90-degree position, and constant 20 N traction was applied to the fingers. Bony landmarks and tendons were recruited to locate the portals, while the dorsal and volar portal sites were marked after being identified by palpation. 

In particular, the 1-2 portal was identified between the tendons of extensor carpi radialis brevis (ECRB) along with extensor carpi radialis longus (ECRL) on the ulnar side and abductor pollicis longus (APL) along with extensor pollicis brevis (EPB) on the radial side, in the dorsal aspect of the snuffbox, bounded distally by the scaphoid and extensor pollicis longus (EPL), while proximally by the styloid process of radius [13, 14]. The 3-4 portal was identified in the soft spot between the tendons of extensor digitorum communis (EDC) on the ulnar side and EPL on the radial side, 1 cm distal to Lister's tubercle, in line with the radial border of the third metacarpal, bounded proximally by the distal aspect of the radius. The 4-5 portal was identified between the tendons of extensor digiti minimi (EDM) on the ulnar side and EDC on the radial side, in line with the fourth metacarpal, distal to 3-4 and 1 cm radial to the 6-R portal, bounded distally by the lunate and proximal by the radius. The 6-R and 6-U portals were identified as just radial and ulnar to the extensor carpi ulnaris (ECU) tendon, respectively, both bounded proximally by the triangular fibrocartilage complex (TFCC) just above the styloid process of the ulna, while 6-R was bound distally by the lunate and 6-U by the triquetrum. The MC-R portal was identified between the tendons of EDC on the ulnar side and ECRB on the radial side, 1cm distal to the 3-4 portal along the axis of the radial border of the third metacarpal.

The MC-U portal was identified between the tendons of EDM on the ulnar side and EDC on the radial side, 1 cm distal to 4-5 portal along the axis of the fourth metacarpal. The volar portal sites were marked after an outside-inside technique was applied. Specifically for the VR portal, a 1.5 cm incision was made in the proximal palmar crease of the wrist, following the retraction of the flexor carpi radialis (FCR) tendon to the ulnar side, exposing the volar capsule. The portal was then identified in the plane between the FCR and the radial artery [9]. For the VU portal, a 1.5-cm incision was made at the ulnar side of the flexor digitorum tendons at the level of the proximal wrist crease, following the retraction of the flexor digitorum superficialis (FDS) and profundus (FDP) tendons to the radial side. The portal was then identified in the plane between the ulnar neurovascular bundle and the flexor tendons [10]. For the volar central portals, a longitudinal incision was made in the axis of the third intermetacarpal space, the distal end of which was the distal palmar wrist crease. Then, the entire mass of the FDS was retracted to the radial side, exposing the FDP tendons. The interval between the third and fourth FDP tendons was identified, while the fourth and fifth FDP tendons retracted toward the ulnar side and the third and second toward the radial side, thus exposing the volar capsule. The VC-RC portal was identified proximal to the lunate and in the interval between the short radiolunate ligament and the ulnocarpal ligaments. Finally, the VC-MC portal was identified just above the anterior horn of the lunate, at the level of Poirier’s space. Subsequently, needles were inserted through the marked portal sites to the radiocarpal and midcarpal joints, forming the wrist arthroscopy portals for the upcoming measurements. The dissection was performed using standard dissection tools and 2.5mm loupe magnification. The skin and subcutaneous tissue were excised to expose tendons, nerve branches, and blood vessels. During the dissection, tendons were retracted radially and ulnarly accordingly, while needles were carefully held in place to assess their correct positioning, perforating the joint capsule for each portal.

The nearest structures considered at risk to dorsal portals included the dorsal carpal branch of the radial artery (RA), the superficial branch of the radial nerve (SBRN), the posterior interosseous nerve (PIN), with its terminal branch located on the floor of the fourth extensor compartment, and the dorsal branch of the ulnar nerve (DBUN) [13-19]. The nearest structures considered at risk to volar portals included the radial artery (RA), the median nerve (MN) and its palmar cutaneous branch (PCBMN), the ulnar artery (UA), and the ulnar nerve (UN) [10-13, 20-25].

The distances were measured as the shortest distance from the needles located in the portals to a corresponding needle placed in the nearest structure at risk, from the ulnar and the radial sides accordingly, in the same anatomical plane of the structure. All measurements were taken by an author using a universal digital caliper (rated accuracy: 0.02mm), and to ensure quality control, they were constantly observed by a different author. The presence of any injury to the structures at risk and the distances to these structures from the portals were recorded, and if a structure was in contact with the needle, it was recorded as 0.00mm.

Especially, dorsal measures were taken from the 1-2 portal to the RA and SBRN, from the 3-4 portal to the SBRN and PIN, from the 4-5 portal to the PIN and DBUN, from the MC-R portal to the SBRN and PIN, from the MC-U portal to the PIN and DBUN, and from the 6-R and 6-U portals to the DBUN. In the same manner, volar measures were taken from the VR portal to the MN, PCBMN, the RA, and the SBRN. Measures in the VU portal were taken from the UA and UN, while measures from VC-RC and VC-MC were taken from the MN, PCBMN, UA, and UN. The measures from the volar portals were made after removing the retractors previously used to identify and mark them.

Statistical analysis of the measurements was performed with the IBM SPSS software version 27 (IBM Corp., Armonk, NY). The normal distribution of the measurements was checked, taking into account the sample size. The quantitative variables of the measurements did not follow a normal distribution; therefore, they were described using the median, the standard error (SE), and the interquartile range (IQR) for continuous variables.

Safe zones, free of neurovascular structures, were defined for all wrist arthroscopy portals that were studied. Correspondingly, for each dorsal and volar portal, the nearest structure at risk was recorded, and then the area surrounding that portal at a distance at least equal to the Q1 value of IQR was considered a safe zone. Afterward, a safety classification for the portals was attempted, ordering them into three classes, from relatively safe to most perilous.

## Results

In this study, there was no injury to blood vessels, nerves and their branches, or extensor and flexor tendons in any of the 29 cadaveric limbs (15 right and 14 left). Median and IQR distances from the 1-2 portal to the RA and SBRN were found to be 2.15±0.26mm (1.10-3.14) and 1.78±0.23mm (1.02-2.43), respectively (Table 1).

**Table 1:** Distances in millimeters (mm) from dorsal and volar portals to structures at risk, in a total of 29 specimens RA: radial artery; SBRN: superficial branch of radial nerve; PIN: posterior interosseous nerve; DBUN: dorsal branch of ulnar nerve; MN: median nerve; PCBMN: palmar cutaneous branch of median nerve; UA: ulnar artery; UN: ulnar nerve

Portal		Structure	Median	Q1 – Q3	SE
Dorsal portals	1 – 2 to:	RA	2.15	(1.10 – 3.14)	± 0.26
		SBRN	1.78	(1.02 – 2.43)	± 0.23
	3 – 4 to:	SBRN	25.99	(23.14 – 29.29)	± 0.95
		PIN	4.10	(2.77 – 5.78)	± 0.38
	4 – 5 to:	PIN	12.33	(9.42 – 15.04)	± 0.69
		DBUN	19.38	(15.33 – 23.08)	± 1.01
	6 – R to:	DBUN	8.26	(5.95 – 11.21)	± 0.61
	6 – U to:	DBUN	2.61	(0.81 – 4.99)	± 0.55
	MC – R to:	SBRN	27.87	(19.81 – 38.15)	± 2.00
		PIN	11.34	(9.50 – 12.95)	± 0.43
	MC – U to:	PIN	16.14	(12.22 – 23.32)	± 1.18
		DBUN	22.95	(16.03 – 29.19)	± 1.56
Volar portals	VR to:	MN	7.73	(5.21 – 10.70)	± 0.71
		PCBMN	3.57	(1.99 – 4.96)	± 0.38
		RA	5.76	(4.35 – 7.06)	± 0.46
		SBRN	17.61	(11.54 – 25.83)	± 1.79
	VU to:	UA	4.25	(3.07 – 6.00)	± 0.36
		UN	7.47	(5.36 – 9.29)	± 0.41
	VC – RC to:	MN	10.33	(7.59 – 13.34)	± 0.57
		PCBMN	16.59	(14.31 – 19.10)	± 0.52
		UA	7.73	(6.25 – 9.40)	± 0.33
		UN	9.15	(7.23 – 11.19)	± 0.42
	VC – MC to:	MN	8.16	(6.17 – 10.02)	± 0.41
		PCBMN	15.66	(13.94 – 17.94)	± 0.53
		UA	5.99	(4.19 – 7.82)	± 0.36
		UN	7.89	(5.39 – 10.29)	± 0.45
Portal		Structure	Median	Q1 – Q3	SE
Dorsal portals	1 – 2 to:	RA	2.15	(1.10 – 3.14)	± 0.26
		SBRN	1.78	(1.02 – 2.43)	± 0.23
	3 – 4 to:	SBRN	25.99	(23.14 – 29.29)	± 0.95
		PIN	4.10	(2.77 – 5.78)	± 0.38
	4 – 5 to:	PIN	12.33	(9.42 – 15.04)	± 0.69
		DBUN	19.38	(15.33 – 23.08)	± 1.01
	6 – R to:	DBUN	8.26	(5.95 – 11.21)	± 0.61
	6 – U to:	DBUN	2.61	(0.81 – 4.99)	± 0.55
	MC – R to:	SBRN	27.87	(19.81 – 38.15)	± 2.00
		PIN	11.34	(9.50 – 12.95)	± 0.43
	MC – U to:	PIN	16.14	(12.22 – 23.32)	± 1.18
		DBUN	22.95	(16.03 – 29.19)	± 1.56
Volar portals	VR to:	MN	7.73	(5.21 – 10.70)	± 0.71
		PCBMN	3.57	(1.99 – 4.96)	± 0.38
		RA	5.76	(4.35 – 7.06)	± 0.46
		SBRN	17.61	(11.54 – 25.83)	± 1.79
	VU to:	UA	4.25	(3.07 – 6.00)	± 0.36
		UN	7.47	(5.36 – 9.29)	± 0.41
	VC – RC to:	MN	10.33	(7.59 – 13.34)	± 0.57
		PCBMN	16.59	(14.31 – 19.10)	± 0.52
		UA	7.73	(6.25 – 9.40)	± 0.33
		UN	9.15	(7.23 – 11.19)	± 0.42
	VC – MC to:	MN	8.16	(6.17 – 10.02)	± 0.41
		PCBMN	15.66	(13.94 – 17.94)	± 0.53
		UA	5.99	(4.19 – 7.82)	± 0.36
		UN	7.89	(5.39 – 10.29)	± 0.45

**Figure 1:**
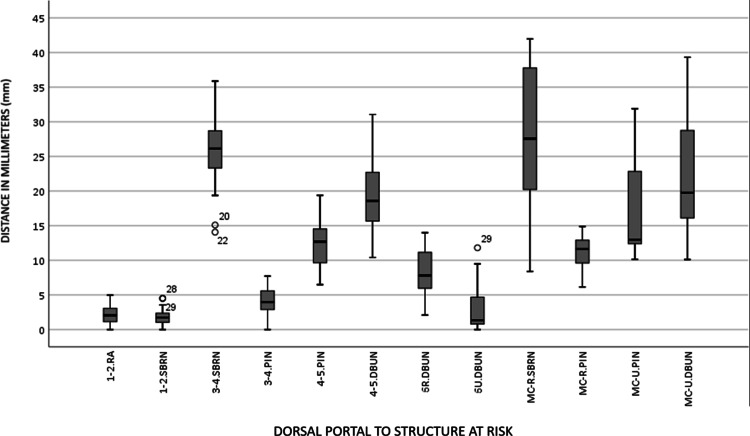
Distances in millimeters (mm) from dorsal portals to structures at risk in a total of 29 specimens are shown in the graph. RA: radial artery; SBRN: superficial branch of the radial nerve; PIN: posterior interosseous nerve; DBUN: dorsal branch of the ulnar nerve; MC-R: midcarpal radial; MC-U: midcarpal ulnar

The distance from important structures varied among dorsal portals, making the risk of surgery significantly different. (Figure 1).

The safe zone surrounding the 1-2 portal was only greater than 1.02mm (Table 2).

**Table 2:** Safety classification of the wrist arthroscopy portals SBRN: superficial branch of radial nerve; PIN: posterior interosseous nerve; DBUN: dorsal branch of ulnar nerve; PCBMN: palmar cutaneous branch of median nerve; UA: ulnar artery

	Dorsal portals				Volar portals		
	Portal	Safe zone	Nearest structure at risk		Portal	Safe zone	Nearest structure at risk
A class	MC – U	12.22 mm	PIN	A class	VC – RC	6.25 mm	UA
	MC – R	9.50 mm	PIN				
	4 – 5	9.42 mm	PIN				
B class	6 – R	5.95 mm	DBUN	B class	VC – MC	4.19 mm	UA
	3 – 4	2.77 mm	PIN		VU	3.07 mm	UA
C class	1 – 2	1.02 mm	SBRN	C class	VR	1.99 mm	PCBMN
	6 – U	0.81 mm	DBUN				
	Dorsal portals				Volar portals		
	Portal	Safe zone	Nearest structure at risk		Portal	Safe zone	Nearest structure at risk
A class	MC – U	12.22 mm	PIN	A class	VC – RC	6.25 mm	UA
	MC – R	9.50 mm	PIN				
	4 – 5	9.42 mm	PIN				
B class	6 – R	5.95 mm	DBUN	B class	VC – MC	4.19 mm	UA
	3 – 4	2.77 mm	PIN		VU	3.07 mm	UA
C class	1 – 2	1.02 mm	SBRN	C class	VR	1.99 mm	PCBMN
	6 – U	0.81 mm	DBUN				

The median and IQR distances from the 3-4 portal to the SBRN and PIN were found to be 25.99± 0.95mm (23.14-29.29) and 4.10± 0.38mm (2.77-5.78), respectively. The safe zone surrounding the 3-4 portal was greater than 2.77mm. The median and IQR distances from 4-5 portals to PIN and DBUN were found to be 12.33±0.69mm (9.42-15.04) and 19.38±1.01mm (15.33-23.08) respectively. The safe zone surrounding the 4-5 portal was greater than 9.42mm. Median and IQR distances from 6-R and 6-U portals to the nearest at-risk DBUN were 8.26±0.61mm (5.95-11.21) and 2.61±0.55mm (0.81-4.99), respectively. The safe zone surrounding the 6-R portal was greater than 5.95mm and only 0.81mm for the 6-U portal. The median and IQR distances from the MC-R portal to SBRN and PIN were 27.87±2.00mm (19.81-38.15) and 11.34±0.43mm (9.50-12.95), respectively. The safe zone surrounding the MC-R portal was greater than 9.50mm. The median and IQR distances from the MC-U portal to PIN and DBUN were 16.14±1.18mm (12.22-23.32) and 22.95±1.56mm (16.03-29.19), respectively. The safe zone surrounding the MC-U portal was greater than 12.22mm.

**Figure 2:**
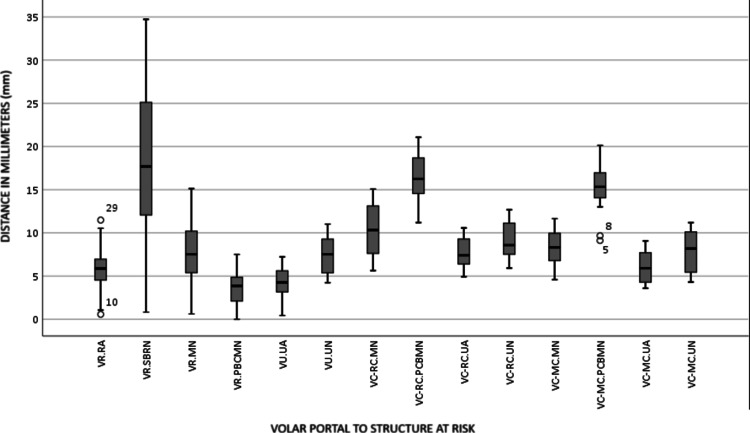
Distances in millimeters (mm) from volar portals to structures at risk in a total of 29 specimens, as shown in the graph VR: volar radial; RA: radial artery; SBRN: superficial branch of the radial nerve; MN: median nerve; PBCMN: palmar cutaneous branch of median nerve; RC: radiocarpal; UA: ulnar artery; VC: volar central; UN: ulnar nerve; MC: midcarpal

Furthermore, the median and IQR distances from the VR portal to the MN, PCBMN, UA, and UN were 7.73±0.71mm (5.21-10.70), 3.57±0.38mm (1.99-4.96), 5.76±0.46mm (4.35-7.06), and 17.61±1.79mm (11.54-25.83), respectively (Figure 2).

The safe zone surrounding the VR portal was greater than 1.99mm. Median and IQR distances from the VU portal to the UA and UN were 4.25±0.36mm (3.07-6.00) and 7.47±0.41mm (5.36-9.29), respectively. The safe zone surrounding the VU portal was greater than 3.07mm. Median and IQR distances from volar central portals to the corresponding structures at risk, MN, PCBMN, UA, and UN, were as follows: For the VC-RC portal: 10.33±0.57mm (7.59-13.34), 16.59±0.52mm (14.31-19.10), 7.73±0.33mm (6.25-9.40), and 9.15±0.42mm (7.23-11.19), respectively; For the VC-MC portal: 8.16±0.41mm (6.17-10.02), 15.66±0.53mm (13.94-17.94), 5.99±0.36mm (4.19-7.82) and 7.89±0.45mm (5.39-10.29), respectively. The safe zone surrounding the VC-RC portal was greater than 6.25mm and 4.19mm for the VC-MC portal.

**Figure 3:**
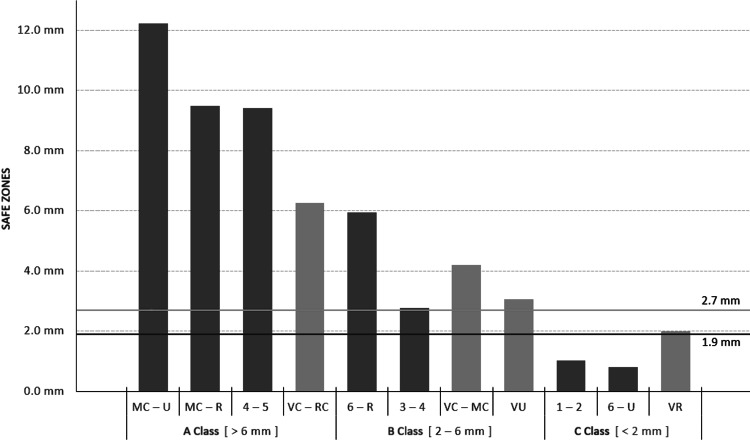
Safety classification of the wrist arthroscopy portals The gray line is the diameter of the standard wrist arthroscope (2.7mm), while the black line is the diameter of the nanoscope (1.9mm). MC-U: midcarpal ulnar; MC-R: midcarpal radial; VC-RC: volar central radiocarpal; VC-MC: volar central midcarpal; VU: volar ulnar; VR: volar radial

Considering the safe zone for each portal and the possible damage caused by the placement of the arthroscopy instruments, three categories for wrist arthroscopy portals were suggested. Portals under Class A can be utilized with sufficient safety, implementing all types of arthroscopes since the nearest structure at risk is located further than 6mm. In Class B, arthroscopy approaches should be considered of average safety because structures at risk are located at a distance between 2mm and 6mm. The least safe portals were classified as Class C, with structures at risk closer than 2mm (Figure 3).

More specifically, in Class A, the dorsal portals were attributed in the following order: the MC-U with a safe zone of 12.22mm, the MC-R with a safe zone of 9.50 mm, and the 4-5 with a safe zone of 9.42mm. Correspondingly, the VC-RC with a 6.25mm safe zone was placed from the volar portals. In Class B, the dorsal portals were placed in the following order: the 6-R with a safe zone of 5.95mm and the 3-4 with a safe zone of 2.77mm. Similarly, the volar portals were attributed in the following order: the VC-MC with a safe zone of 4.19mm and the VU with a safe zone of 3.07mm. Finally, in Class C, the dorsal portals were placed in the following order: the 1-2 with a safe zone of 1.02mm and the 6-U with a safe zone of 0.81mm. Respectively, the VR with a 1.99mm safe zone was attributed to the volar portals.

## Discussion

Wrist arthroscopy is an efficient and minimally invasive technique with low complication rates and good clinical results [26]. It progressively allows wrist surgeons to perform more complex surgeries, expanding the current indications and overcoming challenges [25-28].

Following this study, 1-2 and 6-U are the most perilous dorsal portals and were placed in Class C. Specifically, in their studies, Abrams et al. [27], Kiliç et al. [15], Tryfonidis et al. [28], and Shyamalan et al. [29] found equally small measurements for the SBRN regarding 1-2 portal (3mm/1-6, 5mm/2-12, 3mm/0-19, and 2mm/0-8, respectively), with the last two recording several zero measures. Moreover, this study confirms the findings of Abrams et al. [27] regarding the distance from the 1-2 portal to the RA (3mm/1-5). Considering the 6-U portal, Abrams et al. [27], Ehlinger et al. [18], and especially Shyamalan et al. [29], and Tryfonidis et al. [28] found equally small measurements for the DBUN in their studies (5mm/2-12, 5mm/1-12, 1mm/0-8, and 3mm/0-11, respectively).

According to our study, 6-R and 3-4 are relatively safe dorsal portals and were placed in Class B. In their studies, Abrams et al. and Shyamalan et al. found similar distances from 3-4 portal to SBRN (16mm/5-22 and 25mm/15-33, respectively), while smaller distances were presented by Kiliç et al. and Tryfonidis et al. (9mm/2-19 and 9mm/0-48, respectively) [27-29]. Furthermore, this study confirms Shyamalan et al. and Pan & Hung's finding regarding the distance from the 3-4 portal to the PIN (4.4mm/0-10 and 4.8mm/1.2-12, respectively), while Cheah et al. observed damage to the PIN in 50% of their specimens [16,17,29]. Considering the 6-R portal, Abrams et al. and Shyamalan et al. found similar measurements for the DBUN in their studies (8mm/0-14 and 8mm/2-14, respectively), with Ehlinger et al. measuring smaller distances (4mm/1-15) and Tindall et al. proposing a region as a safe zone within our measurements [18, 19, 27, 29].

The safest dorsal portals, the midcarpals and 4-5 portals, were placed in Class A. Similar distances from the MC-R portal to the SBRN were found by Abrams et al. [27] and Shyamalan et al. [29] (16mm/5-26 and 24mm/13-42, respectively), while Tryfonidis et al. [28] and Kiliç et al. [15] presented smaller distances (10mm/1-20 and 8mm/1-16, respectively) without observing the damage to the nerve in any of the studies. However, Shyamalan et al. and Pan & Hung, in their studies, found distances from the MC-R portal to the PIN (13mm/0-20 and 7.3mm/0-13, respectively), implying possible damage to the nerve [16, 29]. Considering the MC-U portal, in their studies, Abrams et al. [27] and Shyamalan et al. [29] found similar measurements for the DBUN (15mm/4-25 and 25mm/9-56, respectively), while no studies measured the distance from the MC-U portal to the PIN. Regarding distances from the 4-5 portal to the PIN, Shyamalan et al. found similar measurements (12.6mm/2-25), while Pan & Hung presented smaller distances (9mm/3.8-12.7) in their studies [16, 29]. Additionally, this study confirms the findings of Shyamalan et al. and Tryfonidis et al. regarding the distance from the 4-5 portal to the DBUN (21mm/13-32 and 18mm/9-27, respectively), yet Ehlinger et al. found smaller distances in their study (4mm/1-11) [18, 28-29].

Further in this study, VR is the most unsafe volar portal and was placed in Class C. Specifically, Slutsky, in his study, following the same outside-inside technique, found similar distances from the VR portal to the MN, PCBMN, UA, and UN (8mm/6-10, 4mm/3-5, 5.8mm/4-6, and 15.6mm/12-19, respectively) [9]. On the contrary, Gillis & Kakar, with an inside-outside technique, found smaller distances regarding the MN and PCBMN (1mm/0-35 and 1.9mm/0-3, respectively), with injury to the nerves in some specimens, and greater distances regarding the RA and SBRN (13.8mm/9.5-17 and 21mm/13.5-25, respectively) [21].

Moreover, in our study, VC-MC and VU portals were placed in Class B, as they are relatively safe volar portals. In their study, Corella et al., utilizing the same outside-inside technique, found similar distances from the VC-MC portal to the MN, PCBMN, and ulnar neurovascular bundle (7mm/4.8-10.3, 16mm/14.8-19 and 4.5 mm/3.8-9, respectively) [12]. Considering the VU portal, Gillis & Kakar, with an inside-outside technique, found similar distances regarding the UA and UN (3.7mm/0-7 and 8.4mm/5-13, respectively), while Slutsky [10, 21] in his study, determined a related safe zone at 5mm.

Finally, in this study, the VC-RC portal was placed in Class A as the safest volar portal. Specifically, in their study, Corella et al., utilizing the same outside-inside technique, found similar distances from the VC-RC portal to the MN, PCBMN, and ulnar neurovascular bundle (10.5mm/7.8-15, 18.5mm/15.8-20.3, and 7mm/5-10.5, respectively) [12]. 

Nonetheless, this study posed several limitations, considering no arthroscopy instruments were used. Thus, it was attempted to mitigate this with the fact that all measurements were taken practically from the center of arthroscopy portal sites, and the proposed classification for each portal can always be applied with a simultaneous width evaluation of current arthroscopy tools, whether for example, the standard 2.7mm wrist arthroscopy or the newly implemented 1.9mm nanoscope [26].

## Conclusions

Our cadaveric study examined an adequate number of specimens with the described method, proposing our safety classification for the wrist arthroscopy portals. Applying this safety classification to arthroscopic practice, wrist arthroscopy can be performed with fewer iatrogenic complications arising from the implementation of the wrist portals. Pathology may dictate the appropriate wrist portal, and therefore, using the most suitable portal should be done with caution, keeping in mind our classification system. We highly recommend doctors adopt their classification in their everyday practice. Future prospective studies in surgical patients may validate our results.

## References

[1] Bain GI, Munt J, Turner PC (2008). New advances in wrist arthroscopy. Arthroscopy.

[2] Jones CM, Grasu BL, Murphy MS (2015). Dry wrist arthroscopy. J Hand Surg Am.

[3] Munaretto N, Hinchcliff K, Dutton L, Kakar S (2022). Is wrist arthroscopy safer with the nanoscope?. J Wrist Surg.

[4] Ogut E, Yildirim FB, Urguden M, Oruc F, Oguz N (2020). Abnormal type III fusion between lunate and triquetrum: a case report. Int J Surg Case Rep.

[5] Yao J, Osterman AL (2005). Arthroscopic techniques for wrist arthritis (radial styloidectomy and proximal pole hamate excisions). Hand Clin.

[6] Teng XF, Yuan HZ, Chen H (2022). Evaluation of the efficacy of wrist arthroscopic surgery for aseptic necrosis of lunate bone. Orthop Surg.

[7] Shi H, Lu P, Yu D (2022). The training of wrist arthroscopy. Front Med (Lausanne).

[8] Grechenig W, Peicha G, Fellinger M, Seibert FJ, Weiglein AH (1999). Anatomical and safety considerations in establishing portals used for wrist arthroscopy. Clin Anat.

[9] Slutsky DJ (2002). Wrist arthroscopy through a volar radial portal. Arthroscopy.

[10] Slutsky DJ (2004). The use of a volar ulnar portal in wrist arthroscopy. Arthroscopy.

[11] Slutsky DJ (2002). Volar portals in wrist arthroscopy. J Hand Surg.

[12] Corella F, Ocampos M, Cerro MD, Larrainzar-Garijo R, Vázquez T (2016). Volar central portal in wrist arthroscopy. J Wrist Surg.

[13] Root CG, London DA, Schroeder NS, Calfee RP (2013). Anatomical relationships and branching patterns of the dorsal cutaneous branch of the ulnar nerve. J Hand Surg Am.

[14] Tsu-Hsin Chen E, Wei JD, Huang VW (2006). Injury of the dorsal sensory branch of the ulnar nerve as a complication of arthroscopic repair of the triangular fibrocartilage. J Hand Surg Br.

[15] Kiliç A, Kale A, Usta A, Bilgili F, Kabukçuoğlu Y, Sökücü S (2009). Anatomic course of the superficial branch of the radial nerve in the wrist and its location in relation to wrist arthroscopy portals: a cadaveric study. Arthroscopy.

[16] Pan Y, Hung LK (2016). The course of the terminal posterior interosseous nerve and its relationship with wrist arthroscopy portals. J Wrist Surg.

[17] Cheah AE, Le W, Yao J (2017). Incidence of posterior interosseous nerve trauma during creation of the 3-4 wrist arthroscopy portal in cadavers. Arthroscopy.

[18] Ehlinger M, Rapp E, Cognet JM, Clavert P, Bonnomet F, Kahn JL, Kempf JF (2005). Transverse radioulnar branch of the dorsal ulnar nerve: anatomic description and arthroscopic implications from 45 cadaveric dissections (Article in French). Rev Chir Orthop Reparatrice Appar Mot.

[19] Tindall A, Patel M, Frost A, Parkin I, Shetty A, Compson J (2006). The anatomy of the dorsal cutaneous branch of the ulnar nerve - a safe zone for positioning of the 6R portal in wrist arthroscopy. J Hand Surg Br.

[20] Esplugas M, Lluch A, Garcia-Elias M, Llusà-Pérez M (2014). How to avoid ulnar nerve injury when setting the 6U wrist arthroscopy portal. J Wrist Surg.

[21] Gillis JA, Kakar S (2019). Volar midcarpal portals in wrist arthroscopy. J Hand Surg Am.

[22] Xie RG, Xing SG, Tang JB (2015). New procedures for precisely establishing volar wrist arthroscopic portals. J Hand Surg Eur Vol.

[23] Koroglu M, Ertem K, Aslanturk O (2021). The role of arthroscopy in the treatment of common wrist disorders: A retrospective clinical study. Ann Med Res.

[24] Atlan F, Pritsch T, Tordjman D, Khabyeh-Hasbani N, Halperin D, Factor S (2022). Wrist arthroscopy for diagnosis and treatment of acute and chronic conditions. SICOT J.

[25] Chloros GD, Wiesler ER, Poehling GG (2008). Current concepts in wrist arthroscopy. Arthroscopy.

[26] Oh C, Kakar S (2022). Nanoscope arthroscopy: lessons learned in the first 75 cases. J Wrist Surg.

[27] Abrams RA, Petersen M, Botte MJ (1994). Arthroscopic portals of the wrist: an anatomic study. J Hand Surg Am.

[28] Tryfonidis M, Charalambous CP, Jass GK, Jacob S, Hayton MJ, Stanley JK (2009). Anatomic relation of dorsal wrist arthroscopy portals and superficial nerves: a cadaveric study. Arthroscopy.

[29] Shyamalan G, Jordan RW, Kimani PK, Liverneaux PA, Mathoulin C (2016). Assessment of the structures at risk during wrist arthroscopy: a cadaveric study and systematic review. J Hand Surg Eur Vol.

